# Methods for the Determination of Transition Metal Impurities in Cyclotron-Produced Radiometals

**DOI:** 10.3390/ph15020147

**Published:** 2022-01-26

**Authors:** Viktória Forgács, Anikó Fekete, Barbara Gyuricza, Dániel Szücs, György Trencsényi, Dezső Szikra

**Affiliations:** 1 Department of Medical Imaging, Division of Nuclear Medicine and Translational Imaging, Faculty of Medicine, University of Debrecen, Nagyerdei krt. 98., H-4032 Debrecen, Hungary; fekete.aniko@science.unideb.hu (A.F.); gyuricza.barbara@med.unideb.hu (B.G.); szucs.daniel@science.unideb.hu (D.S.); trencsenyi.gyorgy@med.unideb.hu (G.T.); szikra.dezso@med.unideb.hu (D.S.); 2 Faculty of Science and Technology, Doctoral School of Chemistry, University of Debrecen, Egyetem tér 1, H-4032 Debrecen, Hungary

**Keywords:** radiometal, gallium-68, high-performance liquid chromatography, 4-(2-pyridylazo)resorcinol, xylenol orange, transition metal ions

## Abstract

Cyclotron-produced radiometals must be separated from the irradiated target and purified from other metal impurities, which could interfere with the radiolabeling process. We compared different chromatographic and colorimetric methods to determine the amount of transition metals in radioactive samples. Besides commercially available colorimetric tests, 4-(2-pyridylazo)resorcinol and xylenol orange were used as a non-selective metal reagents, forming water-soluble chelates with most of the transition metals immediately. We compared the applicability of pre- and post-column derivatization, as well as colorimetric determination without separation. The studied chromatographic and colorimetric analyses are not suitable to completely replace atomic spectroscopic techniques for the determination of metal contaminants in radioactive samples, but they may play an important role in the development of methods for the purification of radiometals and in their routine quality control.

## 1. Introduction

With the increasing application of cyclotron-produced radiometals, the determination of non-radioactive metal impurities is becoming more important, since they can reduce the radiolabeling efficiency. Most cyclotron sites do not have dedicated ICP or AAS instruments for the in-house measurement of radioactive samples. External laboratories are not prepared to accept radioactive samples and measurement after total decay of the radioactivity, as it is too slow to provide practical feedback for process optimization. For the routine quality control of radiometal solutions, simple and fast limit tests are desired.

From the broad pool of cyclotron-produced radiometals, gallium-68 is receiving the highest attention. Nowadays, the positron-emitting gallium-68 isotope is obtained mainly from ^68^Ge/^68^Ga generators for the synthesis of radiopharmaceuticals, but the recent increase of generator price and availability issues have motivated the development of different cyclotron production methods using liquid- [1,2,3] and solid targets [4,5,6,7,8,9,10,11]. For some well-established gallium radiotracers, it can be observed that the ^68^Ga-labeled compound is replaced by the ^18^F-labeled analogue due to the higher isotope production yield and longer half-life of the latter in large-scale applications, resulting in a logistical and cost-effective advantage. However, the simple labeling chemistry of gallium-68 facilitates the development of new specific radiopharmaceuticals based on receptor–ligand interaction for different diseases and the broadening application of receptor-targeted radionuclide therapy necessitates the use of ^68^Ga-labeled tracers for therapy monitoring and dosimetry.

The longer half-life (4.042 h) and the lutetium-like coordination chemistry of positron-emitting scandium-44 makes it a potential competitor of gallium-68. Scandium-44 tracers were shown to have more similar biodistribution to lutetium-labelled compounds than gallium tracers [12]. Scandium-44 and gallium-68 can be produced with the proton irradiation of appropriate metal targets (^44^Ca(p,n)^44^Sc [13]; ^68^Zn(p,n)^68^Ga [14]). Several authors developed purification methods for scandium-44 [15] using various resins, and Kilian et al. compared some of these methods [16,17]. Transition metal concentration in the purified samples was determined using ICP-OES or ICP-MS techniques after the decay of the radioactivity.

A great advantage of using ICP for metal determination in radiometal samples is the simultaneous detection of all metals in the low concentration (ppm to ppb) range, as the effect transition metals have on labeling efficiency is cumulative in the case of a non-selective chelator (e.g.,: DOTA). However, not all metals are created equal in complex formation reactions. Oehlke et al. compared the influence of numerous metal ions on the radiolabeling reaction of DOTA with gallium-68 isotope. The most interfering metal ions are Cu^2+^, Fe^3+^, Ga^3+^ and Zn^2+^, similar to [^44^Sc]Sc^3+^ [18].

The main limitation of using atomic spectroscopic techniques is that the measurements have to be performed off-site with significant delay. The high sample volume consumption (up to several mL, depending on the method of sample injection) is a further significant drawback. The sample must be inactive at the time of measurement and must not contain long-lived isotopes that could accumulate on the parts of the instrument. European Pharmacopoea describes GFAAS method for the determination of Zn^2+^ and Fe^3+^, with a limit of 10 µg/GBq for cyclotron-produced gallium-68 [19]. Most PET centers are not equipped to perform these measurements. One approach is to determine the typical cold metal content of radiometal samples during the validation of the isotope production and purification process. This can be acceptable if the produced radiometal is used for labeling in a continuous process. But if centralized supply of cyclotron-produced radiometal is performed by a manufacturer and the radiometal for labeling is used elsewhere, the produced solution must be tested for metal content. Countless sources of metals can be found in every laboratory, and trace metal contamination can easily get into the radiometal production process at various points. Non-filtered laboratory air contains a significant amount of rust- and metal-containing dust particles, while powdered gloves and the rubber plungers of the syringes contain zinc, as well as nylon parts and highly pigmented plastics that are sources of trace metals [20]. Even traces of decorative cosmetics can be sources of Bi^3+^ and Sb^2+^ [21]. Without regular checking of metal content, it is hard to identify and avoid these sources. For the determination of metal content, in-house methods would be preferable.

**Figure 1:**
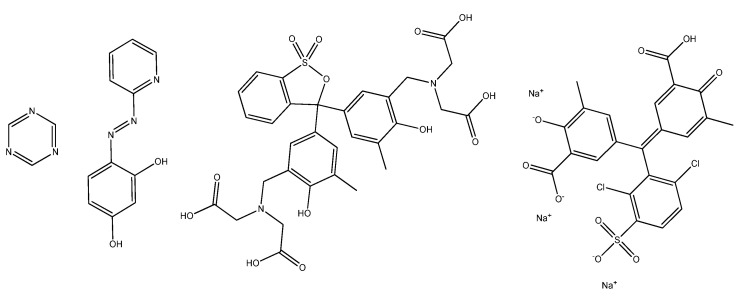
Structure of the applied reagents: triazine, 4-(2-pyridylazo)resorcinol, xylenol-orange, Chromeazurol S.

Besides the well-known atomic spectroscopic techniques, transition metals can also be determined by converting to colored complexes with spectrophotometric or colorimetric techniques with or without chromatographic separation. Complex formation can occur both before and after the column. One of the most widely used complexing agents is 4-(2-pyridylazo)resorcinol (PAR) [22,23] (Figure 1). This tridentate ligand forms colorful water-soluble chelates with most of the transition metals immediately at room temperature. The aqueous solution of PAR is itself colored: red at pH 5.5 or below; orange between pH 6–12.5; and deep red above pH 13.

Post-column derivatization is often used after ion chromatographic separation to quantify metal ions in environmental samples. Transition metals (Fe^3+^, Cu^2+^, Ni^2+^, Zn^2+^, Co^2+^, Cd^2+^, Mn^2+^ and Pb^2+^) can be separated as anionic complexes with pyridine-2,6-dicarboxylic acid (PDCA) containing eluent [24,25,26]. Most often, pyridylazo derivatives (e.g., PAR and 5-Br-PADAP (2-(5-bromo-2-pyridylazo)-5-diethylaminophenol)) are used as complexing agents. The main drawback of the post-column method is the relative complexity of the chromatographic system. Two metal-free HPLC pumps and an injector (autosampler) is necessary. The post-column reagent slowly decomposes in air and contaminates hardware (connections, UV cell). The reagent has to be freshly prepared and flushed through the system prior to measurement and cleared out with careful rinsing of the system.

Besides the post-column method, pre-column complexation is also broadly used. The most important transition metals were examined with: 2-(2-quinolinylazo)-5-diethylaminophenol (QADEAP) in drinking water [27]; with 2-(2-quinolinylazo)-5-dimethylaminophenol (QADMAP) [28] and 2-(8-quinolylazo)-4,5-diphenylimidazole (QAI) [29] in tobacco; with 2-(5-nitro-2-pyridylazo)-5-[*N*-propyl-*N*-(3-sulfopropyl)amino]phenol (nitro-PAPS) [30] in milk powder, wine and drinking water; and with PAR in river water [31].

Transition metal impurities could also be determined by colorimetric tests; for example, xylenol-orange [32,33], triazine [34,35] and Chromeazurol S [36,37] reagents (Figure 1). Colorimetric detection of transition metals can be performed quickly and with high sensitivity in the ppm concentration range, because a complex formation is accompanied by intense color change [38,39].

For the quality control (QC) of radiopharmaceuticals, it is essential to use methods that are reliable and fast. In this report we described the comparison of different chromatographic and colorimetric methods to determine the amount of transition metals in radioactive samples.

## 2. Results and Discussion

### 2.1. Post-Column Derivatization with PAR

**Figure 2:**
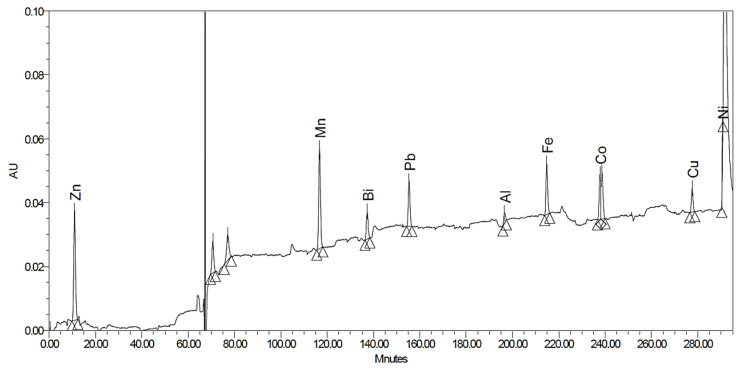
Separation of transition metals with post-column derivatization in test solutions.

Transition metals were separated on a Dionex IonPac CS5A column. The PAR reagent solution was mixed to the effluent to form a light-absorbing complex with the metals. Fe^3+^ and Zn^2+^, considered to be the major contaminants of cyclotron-produced [^44^Sc]Sc^3+^, were baseline separated. Residual zinc ions can be determined down to 0.16 ppm and iron to 0.26 ppm. Besides the two mentioned transition metals, Cu^2+^ ions are also a strong competitor in DOTA-labeling reactions [18]. It cannot be separated from Zn^2+^ ions with the current method, but the presence of both metal ions can be excluded if no peak is observed between 8 and 9 min. Many transition metal ions can be detected with good LOQ values (Table 1). However, the long-term observation of the baseline (Figure 2) shows significant noise, which could be decreased by careful equilibration of the system with the eluents, but it could not be completely avoided.

Thus, small differences in retention times, together with the baseline instability, do not allow unambiguous identification of each peak, however the absence of peaks is a good indication of sample purity.

**Figure 3:**
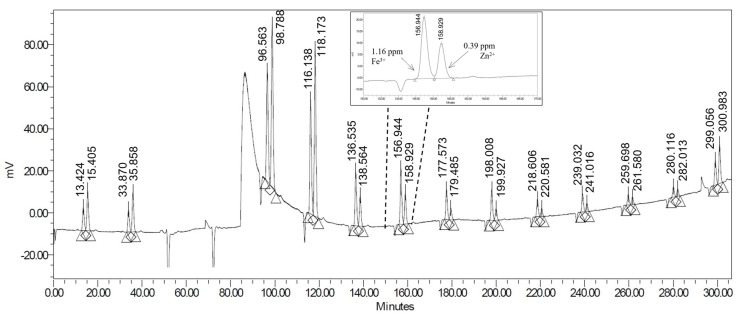
Analysis of radioactive scandium-44 samples on Dionex IonPac CS5A column with PDCA (pyridine-2,4-dicarboxylic acid) eluent.

This method has an acceptable resolution for Fe^3+^ and Zn^2+^ ions (R_s_ = 1.43, Figure 3) under the applied conditions, but for other potentially interfering metal ions (e.g.,: Cu^2+^, Al^3+^) the separation is not appropriate. Therefore, it is able to verify the low metal content of the radiometal samples but does not allow accurate quantification at low concentrations.

The post-column derivatization method is sensitive, but time-consuming to use. In addition, reagents should be freshly prepared by the excluding air before measurement and the system must be carefully equilibrated with the eluents. Despite prolonged washing, significant baseline drift and stability problems were often observed. Based on our results, the use of this method for routine quality control of short-lived isotopes is not recommended.

### 2.2. Pre-Column Derivatization with PAR

**Figure 4:**
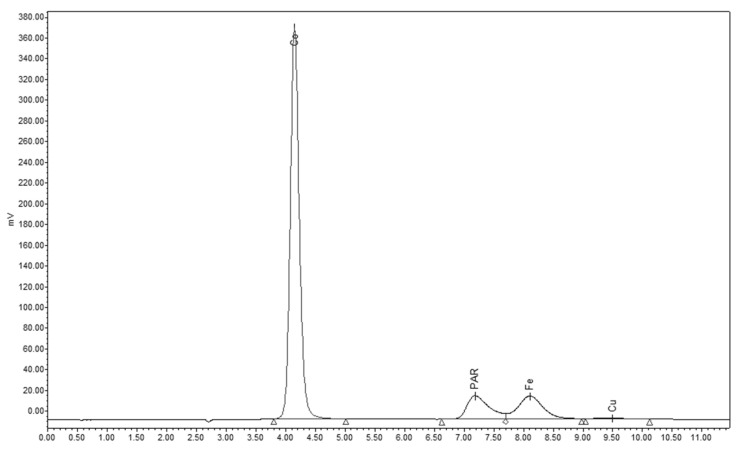
Separation of PAR and its Co^2+^, Fe^3+^ and Cu^2+^ complexes on reversed phase column in test sample.

To avoid the difficulties observed during the post-column derivatization, we applied the same derivatization reaction before sample injection for analysis of the samples. Determination of Co^2+^, Fe^3+^, Cu^2+^ and Zn^2+^ ions were investigated. In the case of Co^2+^, Fe^3+^ and Cu^2+^, the formed metal-PAR complexes were retained on reversed phase column (LiChrospher 100 RP18) and eluted with phosphate buffer–methanol eluent. The unreacted PAR reagent elutes at 6.1 min, and the Fe^3+^-PAR complex was baseline separated (R_S_ = 3.09) at 7.8 min with detection at 530 nm (Figure 4). The calibration curve was linear (R^2^ = 0.998) in the 0.5–10 ppm range. Limit of quantitation was found to be 0.1 ppm.

Zn^2+^ ions could not be determined with pre-column derivatization, as a solid precipitate was formed in the sample which was irreversibly retained on the column. However, this method was suitable for the determination of Fe^3+^ with good sensitivity and in the case of Cu^2+^ contaminants the sensitivity was moderate (Table 2). Results of iron determination was in good agreement with ICP data (Table 3).

### 2.3. Colorimetric Metal Determination

The determination of the small amount of transition metal ions (Fe^3+^, Zn^2+^, Al^3+^) was also investigated by commercially available colorimetric tests. These tests are based on the selective formation of a metal–dye complex and the selectivity of a particular metal ion is often enhanced by masking of interfering metal ions. Therefore, the tests contain several reagents to be added to the sample before the color formation reaction. The main disadvantage of commercially available colorimetric tests is that they require a large volume of sample, which must be reduced when testing radioactive samples. The original color comparison method does not allow the decrease of test solution volume without reducing the sensitivity. This could be overcome by changing the method of color determination from visual to instrumental, using UV-VIS detection. Thus, we decreased the sample and reagent volumes (Table 4) and injected the samples to the HPLC-UV detector, without the presence of a column.

**Figure 5:**
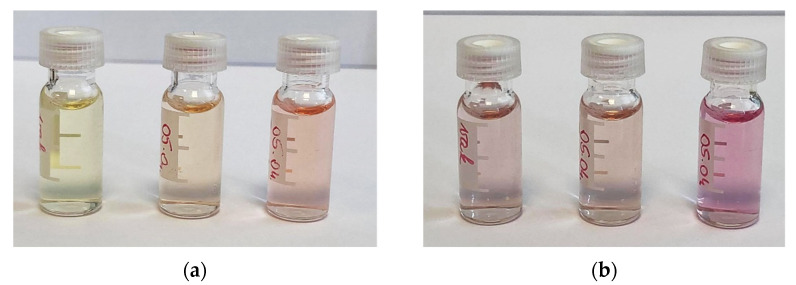
Color change of PAR and xylenol orange solutions in the presence of various amounts of Fe^3+^ and Zn^2+^ ions. (**a**) Colors with PAR at 0, 5 and 10 ppm Fe^3+^; (**b**) Colors with XO at 0, 5 and 10 ppm Zn^2+^.

Besides the colorimetric tests, we investigated the use of PAR- and xylenol orange reagents without separation to determine the metal content of the radioactive samples. The formation and the color of the metal–PAR complex was pH-dependent; thus, the addition of buffer was essential. Accordingly, 1 M ammonium acetate buffer (pH = 6.5) was used to adjust the pH of the samples. The strong orange color of the PAR reagent did not allow the observation of a color change when the PAR concentration of the sample solution was 200 µM, so it was optimized. Using a 10 µM PAR solution, a color change from light yellow to pink was clearly visible (Figure 5). The LOQ was 0.21 ppm for Fe^3+^ with approximately 10 µM PAR, using photometric detection at 490 nm. The calibration curve was linear (R^2^ = 0.9993) up to 10 ppm Fe^3+^ concentration.

Xylenol orange was successfully used for Zn^2+^ determination in samples containing 0.01 M HCl solution (LOQ 0.61 ppm, R^2^ = 0.9996), but not in the case of high hydrochloric acid concentration (2–5 M HCl). Despite the neutralization of samples by large excess of the buffer, the spectra of xylenol orange was shifted, causing a decrease of absorbance on the examined wavelength. This was probably due to the presence of chloride anions. This limits the use of this test for only low HCl-containing samples, so it can be used to test the residual Zn^2+^ content of purified gallium-68 samples, but is not applicable for checking the intermediate samples of the purification. Similar disturbance was observed in the presence of other transition metal ions in the sample (Fe^3+^, Cu^2+^) with a concentration higher than 1 ppm. This should be also taken into consideration when attempting to use xylenol orange for zinc determination. Accordingly, to exclude the presence of Fe^3+^- and other interfering transition metal ions, the PAR test (pre-column derivatization or colorimetric) should be used first, followed by the xylenol orange test to determine Zn^2+^ content of radioactive samples.

The tested colorimetric reagents (Table 5) can be used in the low ppm range for metal determination, but based on our results, the typical Fe^3+^- and Zn^2+^ content of the purified gallium-68 solutions was under the LOQ of the examined methods due to the use of ultrapure chemicals and careful purification.

## 3. Materials and Methods

### 3.1. Chemicals and Reagents

All chemicals (buffers, 2-dimethylaminoethanol, pyridine-2,6-dicarboxylic acid, potassium hydroxide, formic acid, potassium sulfate, ammonium hydroxide, sodium bicarbonate, natural calcium) were obtained from Sigma-Aldrich (Budapest, Hungary) and used without further purification. 4-(2-pyridylazo)resorcinol (PAR) was purchased from Alfa Aesar (Kandel, Germany). DGA, Zr and TK200 resins were purchased from TrisKem (Bruz, France). Enriched zinc-68 (98.60%) was obtained from NeonestAB (Stockholm, Sweden). Ultrapure hydrochloric acid (35%) and ultrapure nitric acid (69%) were supplied by Carl Roth GmbH (Karlsruhe, Germany). Metal ion standard solutions (1000 mg/L Fe^3+,^ Co^2+^, Cu^2+^, Ni^2+^, Bi^3+^ in nitric acid for AAS), colorimetric tests (MColortest Zinc Test 1.14412.0001, Mqant Aluminium Test 1.14413.0001, Mcolortest Iron Test 1.14403.0001) and xylenol orange (XO) were supplied by Merck (Budapest, Hungary). HPLC-grade solvents were purchased from VWR. Ultrapure water was obtained from a Merck Simplicity Water Purification System.

### 3.2. HPLC Equipment and Conditions

#### 3.2.1. Post-Column Complexation

The measurements were performed with a chromatographic system, consisting of a Jasco PU-2080i (metal free) pump, a waters Acquity UPLC BSM pump, a modified Knauer 3800 autosampler (equipped with plastic injector valve, needle, and 10 µL loop) and a Waters 2487 dual λ absorbance detector. A photomultiplier tube (Hamamatsu Photonics H10493-001), equipped with plastic scintillator, was used for the detection of radioactivity. Data were evaluated by Empower 3 chromatography software. Dionex IonPac CS5A (2 × 250 mm) column was used for the separation of transition metals [40]. The eluent contained 7.0 mM PDCA (pyridine-2,6-dicarboxylic acid), 66 mM Potassium hydroxide, 74 mM Formic acid, 5.6 mM Potassium sulfate, delivered with 0.3 mL/min flowrate by the Jasco metal-free pump. The post-column reagent contained 0.5 mM PAR, 1.0 M 2-dimethylaminoethanol, 0.50 M ammonium hydroxide and 0.30 M sodium bicarbonate. It was pumped by the Waters BSM pump to the column effluent with 0.15 mL/min flowrate. Both solutions were prepared and used under an inert atmosphere. Samples were injected without any sample preparation. The separation took 15 min. UV chromatograms were integrated at λ = 530 nm (Table 6).

#### 3.2.2. Pre-Column Complexation

The measurements were performed with a Jasco PU-2080i (metal free) pump, a modified Knauer 3800 autosampler (equipped with plastic valve, needle, and 10 µL loop) and a Waters 2487 dual λ absorbance detector. Flow rate of 0.8 mL/min on a LiChrospher 100 RP18 column (75 × 4 mm, 5 µm). The mobile phase was 65% 0.1 M; pH 6.5 NH_4_H_2_PO_4_/(NH_4_)_2_HPO_4_ buffer and 35% methanol. Samples were mixed in 1:1 ratio with PAR reagent solution, prepared by dissolving 6 mg of solid PAR in 3.5 mL methanol and 6.5 mL NH_4_H_2_PO_4_/(NH_4_)_2_HPO_4_ buffer (0.1 M; pH 6.5). Formation of the PAR complex was accompanied by an immediate color change (Table 6).

#### 3.2.3. Colorimetry

The measurements were performed with a Jasco PU-2080i (metal free) pump, a modified Knauer 3800 autosampler (equipped with plastic valve, needle and 10 µL loop) and a Waters 2487 dual λ absorbance detector. The injector was directly connected to the detector—without column. Eluent was water with 0.8 mL/min flowrate. UV detection was performed at 490 nm (Table 6). Table 4 shows the sample preparation methods for different colorimetric analyzes.

### 3.3. Production and Purification of Scandium-44 and Gallium-68 Radionuclides

Scandium-44 and gallium-68 isotopes were produced in a GE PETtrace cyclotron with proton irradiation of solid calcium and zinc metal targets (^44^Ca(p,n)^44^Sc), (^68^Zn(p,n)^68^Ga) using a shuttle-type solid target system developed in-house [15], equipped with 50 µm HAVAR- and 500 µm aluminum energy degrader foils. The targets were prepared by pressing approximately 120 mg natural calcium metal or 40 mg enriched zinc-68 powder to form a pellet and these pellets were pressed into aluminum target holders. Targets were irradiated with 30–50 µA proton beam for 10–180 min.

Purification of [^44^Sc]Sc^3+^: Irradiated target (with 100–500 MBq activity) was placed in a lead pot and pressed against a plastic dissolution block equipped with an O-ring seal and two Teflon capillaries ending at the target metal surface. The irradiated metal was dissolved in 4 mL 3 M u.p. HCl at room temperature, pumped at 1 mL/min with a syringe pump. Target solution was loaded onto 70 mg DGA resin and washed with 3 mL 3 M HCl (removal of Ca^2+^) and 3 mL 1 M HNO_3_ (removal of Fe^3+^, Zn^2+^, Ni^2+^). [^44^Sc]Sc^3+^ was eluted fractionally with 1 mL 0.1 M HCl. [^44^Sc]Sc^3+^ recovery was 75–85%.

Purification of [^68^Ga]Ga^3+^: Irradiated target (2–60 GBq) was dissolved in 10 mL 5 M u.p. HCl at room temperature in the same dissolution block and loaded to Zr resin, washed with 10 mL 5 M HCl and eluted to TK200 resin with 5 mL 2 M HCl. Final elution from the second resin was performed with 0.05 M HCl.

## 4. Conclusions

We have compared various metal determination methods, utilizing colored complex formations with different dyes.

Based on our studies, chromatographic separation with post-column derivatization is a more selective and sensitive method for trace metal analysis, but the complexity of the instrumental setup and the observed baseline instabilities prohibit its use for routine QC. Furthermore, commercially available or in-house-developed colorimetric tests can be useful for the detection of higher (more than few ppm) concentrations of contaminating metals, but is not selective enough to use in the sub-ppm range, where the typical metal content of radiometal samples can be found. We have found that the reliability and documentation of colorimetric testing can be improved by injecting the samples to the HPLC-UV detector without a column. In addition, the use of HPLC detection enables the use of much smaller sample volumes (30 µL instead of 5–20 mL), which significantly reduces the material loss of short-lived radiometals during QC. The most important advantage of colorimetric methods is the possibility to obtain information about the metal content of a sample without delay, which together with labeling tests (e.g., apparent molar activity determination) can facilitate method development, help in troubleshooting and may enable some level of process control in routine radiotracer production.

The examined colorimetric tests cannot replace atom spectroscopic techniques for the determination of trace metal impurities in radioactive samples but can find their place in purification method development and routine QC of radiometal solutions for labeling.

## Figures and Tables

**Table 1:** Quantitation limits for the examined non-radioactive metals with post-column derivatization.

Metal Ions	LOQ (ppm)	tR (min.)	c (ppm)	Rs
Bi^3+^	8.85	5.32	1.0	-
Fe^3+^	0.26	6.56	0.5	0.89
Zn^2+^	0.16	8.52	0.5	1.43
Cu^2+^	0.13	8.87	0.3	0.26
Ni^2+^	1.39	9.48	1.0	0.80
Al^3+^	1.17	9.69	1.0	0.25
Pb^2+^	0.31	9.75	1.0	0.04
Co^2+^	0.13	9.96	1.0	0.15
Cd^2+^	0.33	10.41	0.3	0.33
Mn^2+^	0.10	17.57	1.0	5.17

**Table 2:** Separation of transition metal ions in test solution.

Metal	LOQ (ppm)	tR (min.)	c (ppm)	Rs
Co^2+^	0.004	4.14	4.91	-
PAR	-	7.19	-	6.54
Fe^3+^	0.1	8.12	3.3	1.33
Cu^2+^	22.33	9.50	52.96	1.78

**Table 3:** Comparison of Fe^3+^ content of gallium-68 samples, determined with ICP (inactive samples) and pre-column derivatization (radioactive samples).

Samples	c(Fe^3+^) ppm	
	ICP	HPLC Pre-Column Derivatization with PAR
07.02	0.39	0.41
07.01/2	0.41	0.30
07.01/1	0.39	0.24

**Table 4:** Sample preparation methods of colorimetric tests.

Reagent	Original Method ^1^	Sample Preparation for HPLC Detection
PAR	-	Reagent: 6 mg PAR was dissolved in 3.5 mL methanol and 6.5 mL buffer ^2^. Sample preparation: 5 µL of PAR solution, 1465 µL of buffer ^1^ and 30 µL sample.
Xylenol orange	-	Reagent: 14.3 mg xylenol orange was dissolved in 10 mL buffer ^1^. Sample preparation: 10 µL of XO solution, 1460 µL of buffer ^1^ and 30 µL sample.
Zinc test	5 mL of sample, 4 drops of reagent 1 (160 µL), 1 dosing spoon of reagent 2 (188 mg) and 1 microspoon of reagent 3 (10.3 mg). Leave to stand for exactly 5 min (reaction time). And add 4 drops of reagent 4 (160 µL).	45 µL of reagent 1, 53 mg of reagent 2, 9 mg of reagent 3, 1380 µL of buffer ^1^, 30 µL sample, leave to stand for exactly 5 min (reaction time), and 45 µL of reagent 4.
Iron test	20 mL of sample and 5 drops of reagent 1 (200 µL). Leave to stand for 3 min (reaction time).	15 µL of reagent, 1455 µL of buffer ^1^ and 30 µL sample. Leave to stand for 3 min (reaction time).
Aluminum test	5 mL of sample, 1 microspoon of reagent 1 (147.6 mg), 1.2 mL of reagent 2 and 4 drops of reagent 3 (160 µL). Leave to stand for 7 min (reaction time).	34.8 mg of reagent 1, 282 µL of reagent 2, 1150 µL of buffer ^1^, 30 µL sample and 38 µL of reagent 3. Leave to stand for 7 min (reaction time).

^1^ Original methods and reagent 1, 2, 3 were provided by Merck with colorimetric test. ^2^ 1 M ammonium acetate buffer, pH 6.5.

**Table 5:** Comparison of the tested methods.

Reagents	Methods	Metal Determined	Range (ppm)	LOQ (ppm)	Interfering Metal Ions
PAR	post-column	Fe^3+^	0.5–1.0	0.08	-
	pre-column	Fe^3+^	1.95–10.0	0.10	-
	colorimetry	Fe^3+^	1.50–11.0	0.21	Cu^2+^, Ni^2+^, Ga^3+^, Bi^3+^, Co^2+^, Cd^2+^
Triazine derivate (Merck)	colorimetry	Fe^3+^	1.50–13.0	0.62	Co^2+^, Cr^3+^, Cu^2+^, Ni^2+^, Pb^2+^
Xylenol Orange	colorimetry	Zn^2+^	1.50–20.0	0.61	Cu^2+^, Ni^2+^, Fe^3+^, Co^2+^, Al^3+^
Thiocyanate (Merck)	colorimetry	Zn^2+^	1.0–20.0	0.20	Cu^2+^, Fe^3+^, Ni^2+^, Pb^2+^,
Chromazurol S (Merck)	colorimetry	Al^3+^	-	0.04	Ag^+^, Co^2+^, Cr^3+^, Cu^2+^, Fe^3+^, Mn^2+^, Pb^2+^, Sn^2+^, Zn^2+^

**Table 6:** Summary of HPLC methods.

System	Column	Eluent	Flow Rate	Reagent	Detection
Post-column complexation	Dionex IonPac CS5A (2 × 250 mm)	Eluent A: 7.0 mM PDCA, 66 mM Potassium hydroxide, 74 mM Formic acid, 5.6 mM Potassium sulfate Eluent B: 0.5 mM PAR, 1.0 M 2-dimethylaminoethanol, 0.50 M ammonium hydroxide and 0.30 M sodium bicarbonate	A: 0.3 mL/min B: 0.15 mL/min	PAR	530 nm
Pre-column complexation	LiChrospher 100 RP18 column (75 × 4 mm, 5 µm)	65% 0.1 M; pH 6.5 NH_4_H_2_PO_4_/(NH_4_)_2_HPO_4_ buffer and 35% methanol	0.8 mL/min	PAR	530 nm
Colorimetry	-	water	0.8 mL/min	PAR	490 nm
				XO	570 nm
				Zinc test	435 nm
				Iron test	560 nm
				Aluminium test	590 nm

## Data Availability

Data is contained within the article.
